# A meta-analysis of resuscitative endovascular balloon occlusion of the aorta (REBOA) or open aortic cross-clamping by resuscitative thoracotomy in non-compressible torso hemorrhage patients

**DOI:** 10.1186/s13017-017-0142-5

**Published:** 2017-07-14

**Authors:** Ramiro Manzano Nunez, Maria Paula Naranjo, Esteban Foianini, Paula Ferrada, Erika Rincon, Herney Andrés García-Perdomo, Paola Burbano, Juan Pablo Herrera, Alberto F. García, Carlos A. Ordoñez

**Affiliations:** 1 Clinical Research Center, Fundación Valle del Lili, Cali, Colombia; 2 Clinical Foianini, Santa Cruz de La Sierra, Bolivia; 3 Virginia Commonwealth University, Richmon, VA USA; 4 Universidad del Valle, Cali, Colombia; 5 Division of Trauma and Acute Care Surgery, Fundación Valle del Lili, Cali, Colombia; 6 Department of Surgery, Brigham and Women’s Hospital, Boston, MA USA; 7 School of Medicine, Universidad Javeriana Cali, Cali, Valle del Cauca Colombia

**Keywords:** Injuries, Non-compressible torso hemorrhage, REBOA, Resuscitation strategies, Traumatic shock, Endovascular procedures

## Abstract

**Background:**

The objective of this systematic review and meta-analysis was to determine the effect of REBOA, compared to resuscitative thoracotomy, on mortality and among non-compressible torso hemorrhage trauma patients.

**Methods:**

Relevant articles were identified by a literature search in MEDLINE and EMBASE. We included studies involving trauma patients suffering non-compressible torso hemorrhage. Studies were eligible if they evaluated REBOA and compared it to resuscitative thoracotomy. Two investigators independently assessed articles for inclusion and exclusion criteria and selected studies for final analysis. We conducted meta-analysis using random effect models.

**Results:**

We included three studies in our systematic review. These studies included a total of 1276 patients. An initial analysis found that although lower in REBOA-treated patients, the odds of mortality did not differ between the compared groups (OR 0.42; 95% CI 0.17–1.03). Sensitivity analysis showed that the risk of mortality was significantly lower among patients who underwent REBOA, compared to those who underwent resuscitative thoracotomy (RT) (RR 0.81; 95% CI 0.68–0.97).

**Conclusion:**

Our meta-analysis, mainly from observational data, suggests a positive effect of REBOA on mortality among non-compressible torso hemorrhage patients. However, these results deserve further investigation.

**Electronic supplementary material:**

The online version of this article (doi:10.1186/s13017-017-0142-5) contains supplementary material, which is available to authorized users.

## Background

Resuscitative endovascular balloon occlusion of the aorta (REBOA) is a procedure that involves placement of an endovascular balloon in the aorta to obtain proximal control of hemorrhage [1]. In recent years, REBOA has become increasingly popular amongst trauma surgeons [2] for the management of traumatic non-compressible torso hemorrhage (NCTH) [3–5]. Although the use of REBOA in the trauma setting has been studied, most of the information comes from series of patients with blunt or penetrating trauma [6] and clear evidence on its effectiveness for improving mortality rates is lacking. Furthermore, little is known about the comparative effectiveness of REBOA and resuscitative thoracotomy (RT) on mortality in NCTH trauma patients.

The objective of this systematic review and meta-analysis was to determine the effect of REBOA, compared to RT, on mortality among NCTH trauma patients.

## Methods

This systematic review was conducted following Cochrane recommendations [7] and PRISMA guidelines [8].

Our PICO strategy was as follows: Patients: patients with non-compressible torso hemorrhage, intervention: REBOA, comparison: resuscitative thoracotomy, and outcomes: mortality and REBOA deployment complications. For this strategy, the proposed systematic review will answer the following questions:

1. In patients with non-compressible torso hemorrhage, does REBOA in comparison with RT result in reduced mortality?
2. What is the type and frequency of complications related to REBOA deployment in included studies?

### Inclusion criteria

We included studies involving patients that suffered blunt or penetrating injuries to the torso. Studies were eligible if they assessed the effect of resuscitative endovascular balloon occlusion of the aorta (REBOA) on relevant outcomes among NCTH patients and compared it to traditional open aortic occlusion by resuscitative thoracotomy. Studies without comparison group were excluded.

### Outcomes

Mortality and complications related to REBOA deployment were the outcomes of interest. Only those studies with sufficient information on the outcomes (effect size and associated precision) were included in the meta-analysis.

### Search methods

We built a highly sensitive search strategy (search strategies are available in Additional file 1) following established recommendations [9, 10]. The literature search was performed in MEDLINE and EMBASE from inception to May 2017 using and combining terms and synonyms related to our condition of interest (trauma, injuries, non-compressible torso hemorrhage, etc.) and our intervention of interest (resuscitative endovascular balloon occlusion of the aorta). We also searched key journals (Journal of Trauma and Acute Care Surgery, World Journal of Emergency Surgery, European Journal of Trauma and Emergency Surgery and the Scandinavian Journal of Trauma Resuscitation and Emergency Surgery). Finally, references from previous relevant narrative and systematic reviews were examined.

### Study selection and data collection

Two individuals independently examined the titles and abstracts identified in the searches. Articles that appeared relevant were selected for full-text review. Two researchers independently reviewed full-text articles for final eligibility. A third reviewer, an experienced trauma surgeon of our review team, resolved disagreements in both phases. The following information was independently extracted using a standardized data form: author, year of publication, the region of origin, trauma characteristics, patient demographics and clinical characteristics, type of operative interventions performed, number and type of complications, mortality, and measures of association for mortality reported.

### Risk of bias

The internal validity of each non-randomized study included in this systematic review was critically evaluated for bias according to the Methodological Index for Non-randomized studies (MINORS) [11]. MINORS evaluate 12 methodological items by scoring each one as 0 (red) if not reported (high risk of bias); 1 (yellow), reported but inadequate (unclear risk of bias); and 2 (green), reported and adequate (low risk of bias). Moreover, we evaluated three additional domains through which bias can be introduced in a study and that are important in trauma outcomes research. Those were as follows: the risk of indication bias, the risk of survival bias, and the risk of reporting bias (selective reporting) [7, 12]. Two independent investigators made the evaluation of the risk of bias as previously mentioned and computed a graphic representation of potential bias in a visual table where high, unclear, and low risks of bias were represented by the colors red, yellow, and green, respectively.

### Data analysis

A meta-analysis was performed to assess the effect of REBOA on mortality, compared it to open aortic cross-clamping by resuscitative thoracotomy. The meta-analysis was restricted to available measures of associations with their correspondent confidence intervals (CIs).

Unadjusted ORs and their CIs were calculated in a 2 × 2 table using data provided in studies. Adjusted ORs and CIs were extracted as provided. Unadjusted and adjusted odds ratios were pooled using a random-effects (DerSimonian and Laird) meta-analysis. The results were reported in forest plots of the estimated effects of the included studies with a 95% confidence interval (95% CI). Heterogeneity was evaluated using the *I* ^*2*^ test, which corresponded to low (*I* ^2^ < 25%), medium (*I* ^2^ = 25–75%), and high (*I* ^2^ > 75%) heterogeneity.

### Sensitivity analysis

The risk ratio is thought to be a better measure of effect to communicate research findings [13]. Moreover, the odds ratio may overestimate a risk association or a treatment effect when the outcome of interest is common in the study population [14, 15]. In this case, our outcome of interest was mortality, which is a very common outcome in patients suffering severe torso trauma and NCTH. Therefore, we decided to convert adjusted odds ratios (ORs) extracted from the studies to risk ratios (RRs) using an inverse probability weighted binomial model [16]. The model used the following equation to convert an OR to an RR:

$$ \mathrm{R}\mathrm{R}=\frac{\mathrm{OR}}{\left[1\hbox{-} \left( q\hbox{-} \left(\mathrm{OR}\times q\right)\right)\right]} $$

In this equation, *q* is the incidence of the outcome of interest in the unexposed (control group). For this analysis, the unexposed were those groups who underwent open aortic cross-clamping by resuscitative thoracotomy. The transformed measures of effect were pooled using a random effect model.

All analyses were done in Stata statistical software.

## Results

**Fig. 1:**
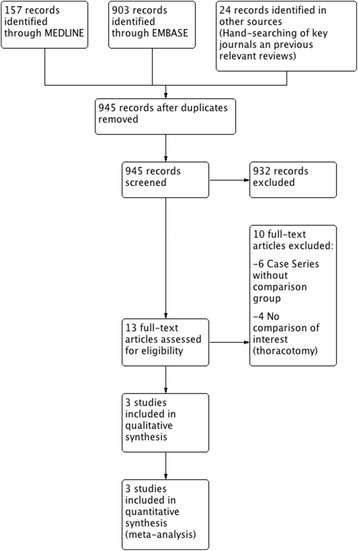
Flowchart according to PRISMA guidelines

We identified 1084 records from our searches, of which 13 studies were eligible to be included in our systematic review. These articles were retrieved as full texts and reviewed. After applying inclusion and exclusion criteria, three studies were included in the systematic review, all of them in both the qualitative and quantitative synthesis (meta-analysis). Figure 1 shows the flowchart for the selection of the studies.

### Characteristics of included studies

Included studies were published in 2016 [17, 18] and 2017 [19]. These studies included a total of 1276 patients. Two were retrospective cohort studies [17, 19] and one was a prospective cohort [18].

### Characteristics of participants

A total of 1276 participants were analyzed in the included studies. Overall, REBOA was deployed in 873 (68%) patients while open aortic cross-clamping by resuscitative thoracotomy was performed in 403 (32%).

An overview of patients’ characteristics by interventions is presented in Table 1. The majority was male (*n* = 851/1276), and all were victims of torso trauma by blunt or penetrating mechanisms. Age structure was heterogeneous and included both young and elderly patients. Two studies reported information on injury severity. In these studies, patients suffered severe trauma and presented primarily with moderate (AIS = 2) and/or serious (AIS = 3) injuries.

**Table 1:** Characteristics of participants

	DuBose 2016		Abe 2016		Aso 2017 ^a^	
	REBOA (*n* = 46)	RT (*n* = 68)	REBOA (*n* = 636)	RT (*n* = 267)	REBOA (*n* = 191)	RT (*n* = 68)
Age ^a^	43.2 (19.6) ^b^	39.2 (16.7) ^b^	52.5 (21.2) ^b^	56.7 (21.1) ^b^	15–49 years = 75	15–49 years = 26
					50–79 years = 95	50–79 years = 35
					≥80 years = 21	≥80 years = 7
Gender (male), *n* (%)	32 (69.6%)	60 (88.2%)	417 (66%)	194 (73%)	114 (59.7%)	44 (64.7%)
SBP	23 (105) ^c^	0(80) ^c^	89 (46) ^c^	87 (45) ^c^	NR	NR
Hypotension (SBP <90), *n* (%)	21 (45.6%)	47 (69.1%)	NR	NR	NR	NR
Trauma mechanism						
Blunt, *n* (%)	35 (76.1%)	36 (53.9%)	591 (93%)	247 (93%)	NR	NR
Penetrating, *n* (%)	11 (23.9%)	32 (47.1%)	45 (7%)	20 (7%)	NR	NR
Cardiac arrest on admission, *n* (%)	16 (34.8%)	35 (51.5%)	212 (33%)	216 (81%)	42 (22%)	42 (61%)
TRISS (PS)	NR	NR	0.43 (0.36) ^b^	0.27 (0.30) ^b^	NR	NR
Injury severity						
ISS	31 (30) ^c^	31.5 (22) ^c^	34 (25) ^c^	34 (20) ^c^	NR	NR
Head AIS	2.0 (5) ^b^	1.5 (4) ^b^	3.6 (1.2) ^b^	3.3 (1.1) ^b^	NR	NR
Chest AIS	1.0 (4) ^b^	3.0 (4) ^b^	3.8 (0.9) ^b^	4.3 (1.1) ^b^	NR	NR
Abdomen AIS	2.5 (3) ^b^	2.0 (5) ^b^	3.6 (1.1) ^b^	3.8 (1.5) ^b^	NR	NR
Procedures						
Laparotomy, *n* (%)	25 (54.3%)	40 (58.8%)	301 (47%)	99 (37%)	17 (8.9%)	12 (17.6%)
Embolization, *n* (%)	3 (6.5%)	3 (4.4%)	153 (24%)	18 (6.7%)	76 (39.8%)	18 (26.5%)
Surgery for liver injury, *n* (%)	7 (15%)	16 (23%)	NR	NR	14 (7.3%)	5 (7.4%)
Spleen resection, *n* (%)	4 (8.7%)	7 (10.3%)	NR	NR	18 (9.4%)	9 (13.2%)
Operative procedures for pelvic fractures	PP 3 (6.5%); PB 4 (8.7%); PEF 3 (6.5%)	PP 10 (14.7%); PB 4 (5.9%); PEF 0 (0%)	NR	NR	OS 15 (7.9%); CS 4 (2.1%)	OS 7 (10.3%); CS 3 (4.4%)
In-hospital mortality, *n* (%)	33 (71.7%)	57 (83.8%)	405 (67%)	210 (90%)	90 (47.1%)	48 (70.6%)
Adjusted measures of association for mortality (REBOA vs. thoracotomy) ^d^	OR 0.263 (95% CI 0.043–1.609) ^e^		OR 0.261 (95% CI 0.130–0.523) ^f^		HR 0.94 (95% CI 0.60–1.48) ^g^	

*SBP* systolic blood pressure, *NR* not reported, *PS* probability of survival, *ISS* Injury Severity Score, *AIS* Abbreviate Injury Score, *PP* pelvic packing, *PB* pelvic binder, *PEF* pelvic external fixation, *OS* open surgery, *CS* closed surgery, *OR* odds ratio, *HR* hazard ratio

Adjusted by:

^a^Patients were categorized in age groups

^b^Mean (SD)

^c^Median (IQR)

^d^As reported in studies

^e^Not reported

^f^Propensity Score Matching by age, gender, mechanism of injury, cause of injury, transport type, pre-hospital treatment, vital signs at emergency department, and injury severity score

^g^Propensity Score Matching by age, sex, body mass index, etiology, Japanese coma scale, presence of head injury, presence of cardiopulmonary arrest on admission, TMPM (Trauma Mortality Prediction Model), and annual number of patients receiving resuscitative thoracotomy (RT) at each hospital

Data on operative interventions was captured. Overall, 494 (38%) and 271 (21%) patients underwent exploratory laparotomy and arterial embolization, respectively. Two studies reported data on splenic and hepatic procedures; these included different damage control strategies such as packing and resections. The same studies reported information on operative interventions for pelvic fractures management, which included the use of packing, external fixation, and surgery.

### Differences between REBOA and resuscitative thoracotomy patients

Two studies reported data on initial blood pressure and injury severity. In these studies, RT patients were more likely to present with significantly lower values systolic blood pressure (SBP: median (IQR): DuBose: REBOA = 23 (105) vs. RT = 0 (80), *p* = 0.01; Abe: REBOA = 89 (46) vs. RT = 87 (45), *p* < 0.001). However, REBOA and RT patients were similar in severity of injuries as reflected by reported AIS and ISS. One study reported the probability of survival for both groups. This study showed that patients that underwent REBOA had a significantly higher probability of survival on admission (TRISS (probability of survival), mean (SD): Abe: REBOA = 0.43 (0.36) vs. RT = 0.27 (0.30), *p* < 0.001].

Resuscitation strategies, including transfusions requirements, were reported in all studies. Two studies reported transfusions requirements as a continuous variable. In these studies, no significant differences were found regarding the amounts of transfusions in first 24 h. The study by Abe et al. reported the number of patients that required transfusions. They found that a significantly higher proportion of patients required transfusions in the REBOA group (Abe: required transfusion, *n* (%): REBOA = 542/636 (85%) vs. RT = 197/267 (74%), *p* < 0.001].

Among operative interventions, overall, REBOA patients underwent arterial embolization more often than RT patients (arterial embolization, *n* (%): REBOA = 232/873 (26%) vs. RT = 39/403 (9.6%), *p* < 0.01). Finally, mortality was significantly higher in patients that underwent RT (mortality, *n* (%): REBOA = 528/873 (60.4%) vs. RT = 315/403 (78.1%), *p* < 0.01].

### Risk of bias

**Fig. 2:**
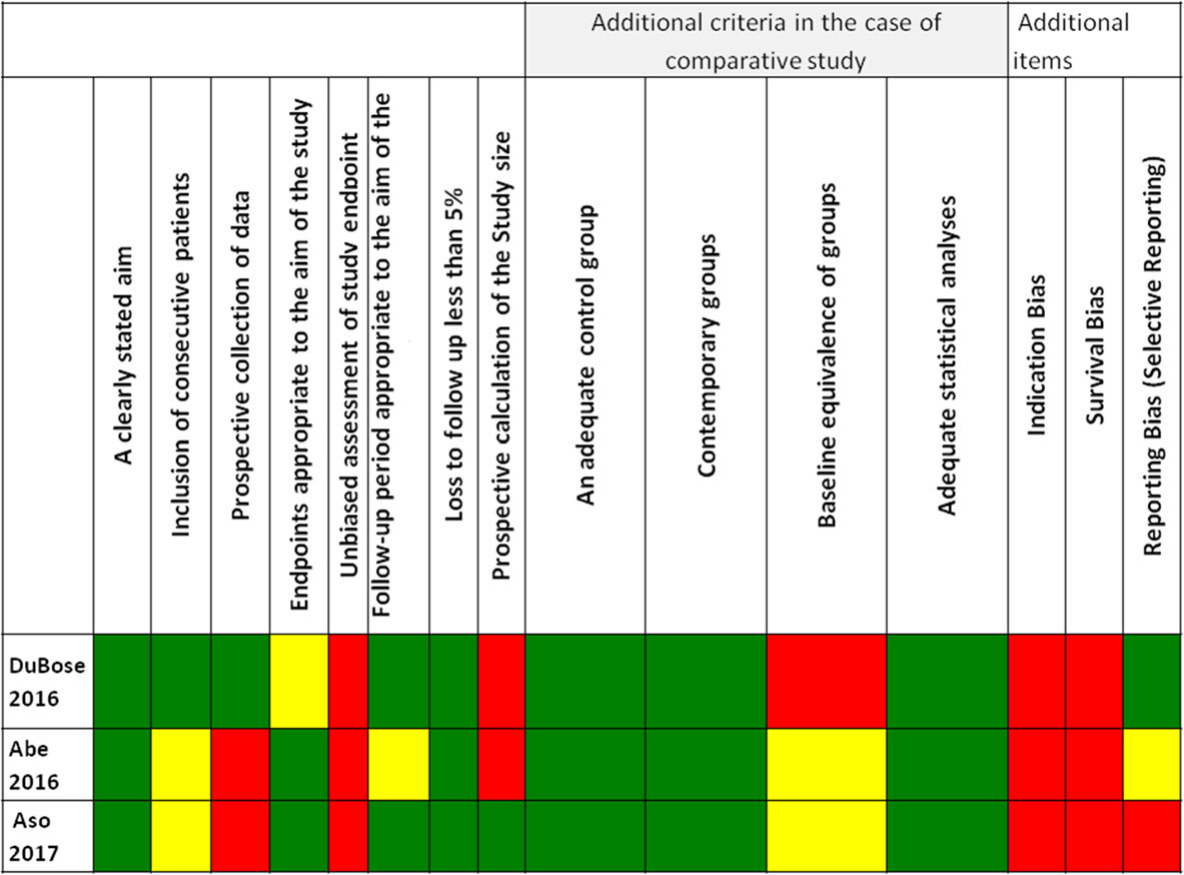
Risk of bias within studies

A summary of the risk of bias is presented in Fig. 2 (a detailed description on how studies were evaluated is available in Additional file 1). Studies were prone to biases present in retrospective studies. However, included studies were rated as having high risk of indication and survival bias. The study by Aso [19] had a high risk of selective reporting.

### Outcomes

#### Mortality

Our primary outcome was mortality. All studies reported information on this outcome. Data captured included the proportion of deaths in each group and the adjusted measures of association for in-hospital mortality. Table 1 provides an overview of the primary outcome information extracted from each study.

### REBOA-related complications

Only the study by DuBose et al. [18] reported complications related to REBOA deployment. In this study, three patients of those in the REBOA group (*n* = 46) suffered complications, which included one pseudoaneurysm and two cases of distal arterial embolism.

### Quantitative synthesis results

**Fig. 3:**
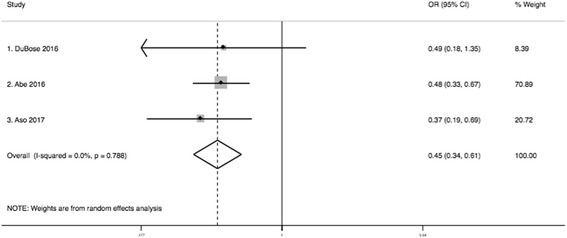
Meta-analysis of unadjusted odds ratios of mortality (REBOA vs RT)

Meta-Analysis of unadjusted odds ratios showed that the odds of mortality were lower in patients that underwent REBOA compared to those that were taken to RT (OR 0.45; 95% CI 0.34–0.61) (Fig. 3).

**Fig. 4:**
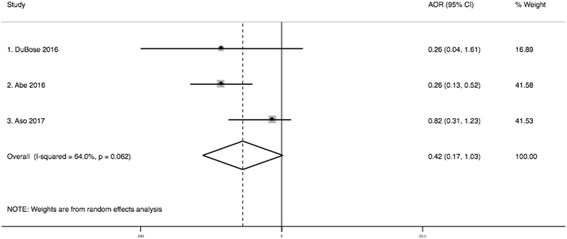
Meta-analysis of adjusted odds ratios of mortality (REBOA vs. RT)

To be able to pool the adjusted odds ratios in a meta-analysis, the hazard ratio reported in the study by Aso [19] was converted to an odds ratio. For the procedure, we assumed that the hazard ratio is a type of relative risk and, thus, is asymptotically similar to a relative risk [14]. Then, using the inverse probability weighted binomial model (10) we transformed the adjusted hazard ratio of mortality reported in the study by Aso [19] to an odds ratio. Following this approach, we obtained an adjusted odds ratio of mortality (Aso: OR 0.821; 95% CI 0.306–1.234). After combining adjusted odds ratios using a random effect model, we found that, although lower in REBOA patients, the odds of mortality did not significantly differ between compared groups (OR 0.42; 95% CI 0.17–1.03) (Fig. 4).

### Sensitivity analyses

When the outcome of interest (mortality) is common, the OR can jeopardize a risk association or a treatment effect by producing biased estimates of the underlying risk ratio (7)(8). In this systematic review, mortality was high in all groups (RT, REBOA) with rates ranging from 47 to 90% (Tables 1 and 2). Therefore, we decided to convert reported ORs to RRs as proposed in the “Methods” section.

**Fig. 5:**
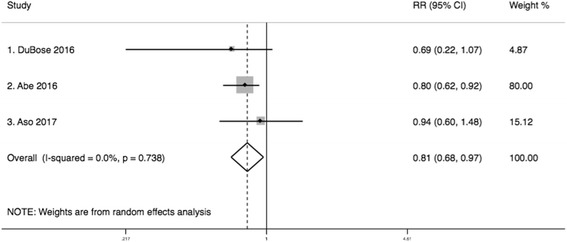
Sensitivity analysis. Meta-analysis of transformed risk ratios of mortality (REBOA vs. RT)

Using the inverse probability weighted binomial model, we obtained the transformed risk ratios of in-hospital mortality for the studies by Abe [17] (RR 0.796; 95% CI 0.624–0.924) and DuBose [18] (RR 0.687; 95% CI 0.217–1.06). For the hazard ratio reported by Aso, we assumed the asymptotical similarity between the hazard ratio and the risk ratio and combined it with the transformed risk ratios using a random effect model. This analysis showed that the risk of mortality was significantly lower among torso trauma patients who underwent REBOA, compared to those who underwent RT (RR 0.81; 95% CI 0.68–0.97) (Fig. 5).

Further sensitivity analyses were performed by methods of adjustment used in individual studies. Therefore, we conducted a meta-analysis of risk ratios from the studies where a propensity score method was used (Aso, Abe) [17, 19]. In this analysis, the risk of mortality was significantly lower in patients who underwent REBOA, compared to those who underwent RT (RR 0.818; 95% CI 0.683–0.979; *I* ^2^ = 0.0%).

A final sensitivity analysis that took into account the evaluation of risk of bias was performed. We ranked the study by Aso [19] with a high risk of selective reporting. They did not report important baseline trauma characteristics such as those related to initial vital signs, physiological status, and injury severity. Not reporting these variables and, furthermore, not including them in the multivariate model analysis could have introduced further indication bias. Therefore, we decided to exclude the study by Aso [19] and to run a random effect model of the remaining studies. This analysis found that the risk of death was significantly lower among REBOA patients (RR 0.79; 95% CI 0.653–0.956; *I* ^2^ = 0.0%).

## Discussion

To our knowledge, this is the first systematic review and meta-analysis assessing the comparative effectiveness of REBOA and resuscitative thoracotomy on mortality among trauma patients suffering NCTH. Our initial analysis found that although lower in REBOA patients, the odds of mortality did not significantly differ between the compared groups. Although sensitivity analyses showed consistent results on the positive effect of REBOA on mortality, results could be comprised by the presence of survival and indication bias within individual studies.

Non-compressible torso hemorrhage (NCTH) is a life-threatening condition defined as shock associated with hemorrhage due to vascular disruption secondary to pulmonary or solid organ injury, major vascular trauma or pelvic fracture [20]. Concerning this, patients included were primarily victims of serious and severe injuries as reflected by the abbreviated injury scale scores and the procedures for hemorrhage control reported. The need for hemostatic procedures is a fundamental component in the definition of NCTH. These comprise mainly open surgery and endovascular interventional procedures. We found that patients included underwent different aggressive surgical and endovascular procedures for immediate hemorrhage control. Therefore, patients analyzed in this systematic review can be classified as suffering wounds that ultimately ended in a pattern of injury consistent with NCTH anatomic definition.

Shock secondary to NCTH is associated with higher mortality rates. However, a proportion of NCTH-related deaths are potentially preventable [20, 21]. Therefore, research on new technologies such as REBOA for further progress in the principles of damage control resuscitation is of paramount importance for improving survival among NCTH patients. Although we found that the risk of death was lower in NCTH REBOA-treated patients, other reports have shown an increased mortality with the use of REBOA in this population. For example, Norii et al. [22] and Inoue et al. [23] showed that among similarly ill trauma patients, the use of REBOA was associated with higher odds of mortality compared to patients who did not receive REBOA.

In this systematic review, REBOA-treated patients underwent endovascular angioembolization more often than RT patients (Arterial embolization, *n* (%): REBOA = 232/873 (26%) vs. RT = 39/403 (9.6%), *p* < 0.01). This result further supports the concept that in patients suffering torso trauma in which NTCH is suspected, early REBOA deployment could aid in hemodynamic stabilization while hemorrhage control is achieved by endovascular procedures. To date, one study reported the use of REBOA followed by arterial embolization [24]. In this study, seven patients with severe hepatic and splenic blunt injuries were managed following damage control resuscitation strategies which included early massive transfusion followed by REBOA deployment for hemodynamic support and angioembolization for definitive hemorrhage control. Six of seven patients survived demonstrating the feasibility of REBOA in these scenarios.

Although our quantitative synthesis shows that REBOA is associated with lower mortality, these results could be flawed by the presence of indication and survival bias within individual studies [12]. Indication bias arises when patients are classified on the basis of the non-randomized intervention they received during the natural course of their medical treatment. Survival bias appears when comparing groups in which patients may die before treatment is initiated [12]. As shown in Table 1, patients that underwent RT were more likely to present with cardiac arrest and lower values of systolic blood pressure. Furthermore, the study by Abe [17] reported that REBOA patients had a significantly higher probability of survival on arrival. These facts could increase the risk of significant indication and survival bias, which are selection biases and are a primary evil in observational studies of non-randomized interventions. The main trouble with these biases is that their presence may produce biased effect estimates, thus comprising validity of results.

Traditionally, RT has been used in severely ill trauma patients as the last effort for resuscitation of the moribund patient [25]. REBOA is thought to emulate RT with the advantage of being a less invasive procedure. However, the fact that RT was performed in patients with a higher physiological exhaustion and with a lower probability of survival illustrates a lack of concrete indications for REBOA use in trauma patients. Moreover, it poses the question if REBOA is a comparable intervention to RT or if it is an intervention aimed to prevent hemodynamic collapse in non-agonal unstable patients.

Knowing the balance of benefits and harms of interventions is a paramount component of evidence-based health care. Therefore, we acknowledge that only the study by DuBose et al. [18] reported complications related to REBOA deployment and that was one main reason to rate the studies by Aso [19] and Abe [17] as having a high and unclear risk of selective reporting respectively. Although the severity and proportion of complications related to REBOA deployment were low in the DuBose series, another report by Saito et al. [26] documented serious adverse events that ultimately ended in lower limb amputations in 2 of 14 patients. However, REBOA is an evolving endovascular technology, and new strategies such as improvements in endovascular skills training [27, 28] and the advent of smaller diameter catheters [29] may improve the safety related to its deployment [30].

Although we found a benefit of REBOA over RT in NCTH patients, our results can be comprised by the biases present in primary studies. Future studies must address specific indications for REBOA to know which population could benefit from its use. Furthermore, trauma researchers should determine if REBOA is a comparable intervention to RT. Therefore, prospective evaluation with specific inclusion and exclusion criteria that ameliorates noise and biased results should be undertaken.

## Conclusion

Our meta-analysis mainly from observational data suggests that the use of REBOA is associated with lower mortality. However, our findings deserve further investigation.

## Additional file

Additional file 1: Search strategy. (DOCX 24 kb)
