# Supplementary material for: A meta-analysis of resuscitative endovascular balloon occlusion of the aorta (REBOA) or open aortic cross-clamping by resuscitative thoracotomy in non-compressible torso hemorrhage patients
Source: World J Emerg Surg. 2017 Jul 14;12:30. doi: 10.1186/s13017-017-0142-5 (PMC5512749; doi:10.1186/s13017-017-0142-5)
Supplement: Additional file 1: — Search strategy. (DOCX 24 kb) [file 13017_2017_142_MOESM1_ESM.docx]

**Additional file 1**

**Search Strategy:**

**MEDLINE:**

1 REBOA.mp.

2 "Resuscitative endovascular balloon occlusion of the aorta".mp.

3 IABO.mp.

4 "intra aortic balloon occlusion catheter".mp.

5 exp Balloon Occlusion/

6 trauma.mp.

7 exp Shock, Hemorrhagic/

8 exp Shock, Traumatic/ or Shock/

9 NCTH.mp.

10 1 or 2 or 3 or 4 or 5 (

11 6 or 7 or 8 or 9

12 10 and 11

**EMBASE**

(reboa or 'resuscitative endovascular balloon occlusion of the aorta' or iabo or 'intra aortic balloon occlusion catheter' or 'balloon occlusion'/exp or 'occlusion balloon catheter'/exp) and ('trauma'/exp or 'traumatic shock'/exp or ncth)

**Risk of Bias explanation:**

The risk of bias of articles included in this systematic review was evaluated using MINORS tool [1]. Additional items that included indication bias, survival bias and reporting bias were evaluated [2,3]. Two individual investigators made a judgment about the possible risk of bias from extracted information, rated as “high risk” (red), “low risk” (green) or “unclear risk” (yellow) of bias. The following table provides and explanation of the evaluation of the risk of bias:

| **Methodological Items** | **Rationale** |
| --- | --- |
| A clearly stated aim | The question addressed should be precise and relevant in the light of available literature |
| Inclusion of consecutive patients | All patients potentially fit for inclusion (satisfying the criteria for inclusion) have been included in the study during the study period (no exclusion or details about the reasons for exclusion) |
| Prospective collection of data | Data were collected according to a protocol established before the beginning of the study |
| Endpoints appropriate to the aim of the study | Unambiguous explanation of the criteria used to evaluate the main outcome which should be in accordance with the question addressed by the study. Also, the endpoints should be assessed on an intention-to-treat basis. |
| Unbiased assessment of the study endpoint | Blind evaluation of objective endpoints and double-blind evaluation of subjective endpoints. Otherwise the reasons for not blinding should be stated |
| Follow-up period appropriate to the aim of the study | The follow-up should be sufficiently long to allow the assessment of the main endpoint and possible adverse events |
| Loss to follow up less than 5% | All patients should be included in the follow up. Otherwise, the proportion lost to follow up should not exceed the proportion experiencing the major endpoint |
| Prospective calculation of the study size | Information of the size of detectable difference of interest with a calculation of 95% confidence interval, according to the expected incidence of the outcome event, and information about the level for statistical significance and estimates of power when comparing the outcomes |
| **Additional criteria in the case of comparative study** | |
| An adequate control group | Having a gold standard diagnostic test or therapeutic intervention recognized as the optimal intervention according to the available published data |
| Contemporary groups | Control and studied group should be managed during the same time period (no historical comparison) |
| Baseline equivalence of groups | The groups should be similar regarding the criteria other than the studied endpoints. Absence of confounding factors that could bias the interpretation of the results |
| Adequate statistical analyses | Whether the statistics were in accordance with the type of study with calculation of confidence intervals or relative risk |
| **Additional Items** | |
| Survival Bias | Is a selection bias that occurs when comparing patient groups in which patients may die before treatment is initiated [3] |
| Indication Bias | It occurs when patients are classified by the nonrandomized intervention they received during the course of their medical treatment overlooking the preceding conditions that necessitated the intervention [3] |
| Selective Reporting | The selective reporting of some outcomes but not others, depending on the nature and direction of the results [2] |

**References**

1. Slim K, Nini E, Forestier D, Kwiatkowski F, Panis Y, Chipponi J. Methodological index for non-randomized studies (MINORS): development and validation of a new instrument . ANZ J. Surg. [Internet]. Blackwell Science Pty; 2003;73:712–6. Available from: http://dx.doi.org/10.1046/j.1445-2197.2003.02748.x

2. Higgins JP, Green S. Cochrane Handbook for Systematic Reviews of Interventions: Cochrane Book Series. Cochrane Handb. Syst. Rev. Interv. Cochrane B. Ser. John Wiley and Sons; 2008.

3. del Junco DJ, Fox EE, Camp EA, Rahbar MH, Holcomb JB. Seven Deadly Sins in Trauma Outcomes Research: An Epidemiologic Post-Mortem for Major Causes of Bias. J. Trauma Acute Care Surg. [Internet]. 2013;75:S97–103. Available from: http://www.ncbi.nlm.nih.gov/pmc/articles/PMC3715063/
